# The role of plasmids in carbapenem resistant *E. coli* in Alameda County, California

**DOI:** 10.1186/s12866-023-02900-2

**Published:** 2023-05-22

**Authors:** Nikolina Walas, Samuel Slown, Heather K. Amato, Tyler Lloyd, Monica Bender, Vici Varghese, Mark Pandori, Jay P. Graham

**Affiliations:** 1 School of Public Health, University of California, Berkeley, CA USA; 2 Alameda County Public Health Laboratory, Oakland, CA USA; 3 Nevada State Public Health Laboratory, Reno, NV USA

**Keywords:** Short-read whole genome sequencing, Carbapenem resistance, ESBLs, *bla*_CTX−M−15_, Plasmids

## Abstract

**Background:**

Antimicrobial resistant infections continue to be a leading global public health crisis. Mobile genetic elements, such as plasmids, have been shown to play a major role in the dissemination of antimicrobial resistance (AMR) genes. Despite its ongoing threat to human health, surveillance of AMR in the United States is often limited to phenotypic resistance. Genomic analyses are important to better understand the underlying resistance mechanisms, assess risk, and implement appropriate prevention strategies. This study aimed to investigate the extent of plasmid mediated antimicrobial resistance that can be inferred from short read sequences of carbapenem resistant *E. coli* (CR-Ec) in Alameda County, California. *E. coli* isolates from healthcare locations in Alameda County were sequenced using an Illumina MiSeq and assembled with Unicycler. Genomes were categorized according to predefined multilocus sequence typing (MLST) and core genome multilocus sequence typing (cgMLST) schemes. Resistance genes were identified and corresponding contigs were predicted to be plasmid-borne or chromosome-borne using two bioinformatic tools (MOB-suite and mlplasmids).

**Results:**

Among 82 of CR-Ec identified between 2017 and 2019, twenty-five sequence types (STs) were detected. ST131 was the most prominent (n = 17) followed closely by ST405 (n = 12). *bla*_CTX−M_ were the most common ESBL genes and just over half (18/30) of these genes were predicted to be plasmid-borne by both MOB-suite and mlplasmids. Three genetically related groups of *E. coli* isolates were identified with cgMLST. One of the groups contained an isolate with a chromosome-borne *bla*_CTX−M−15_ gene and an isolate with a plasmid-borne *bla*_CTX−M−15_ gene.

**Conclusions:**

This study provides insights into the dominant clonal groups driving carbapenem resistant *E. coli* infections in Alameda County, CA, USA clinical sites and highlights the relevance of whole-genome sequencing in routine local genomic surveillance. The finding of multi-drug resistant plasmids harboring high-risk resistance genes is of concern as it indicates a risk of dissemination to previously susceptible clonal groups, potentially complicating clinical and public health intervention.

**Supplementary Information:**

The online version contains supplementary material available at 10.1186/s12866-023-02900-2.

## Background

Antimicrobial resistance (AMR) is a growing concern worldwide. The World Health Organization has estimated that, without intervention, 10 million deaths will occur worldwide by 2050 as a result of AMR infections. The global public health crisis has been further exacerbated by an increase in antibiotic use in response to secondary infection treatment caused by the COVID-19 pandemic, in addition to strained resources for surveillance of hospital-acquired AMR bacterial infections [1]. In the US, extended-spectrum β-lactamase producing *Enterobacterales* attributed approximately 200,000 cases and 10,000 deaths in 2019 [2]. Development of alternative drugs for AMR infections remains challenging and slow [3]. As a result, surveillance is a vital tool for assessing the spread of AMR and monitoring the impact of non-clinical interventions.

β-Lactams are the most widely used group of antibiotics and include penicillins, cephalosporins, monobactams and carbapenems. Resistance has been steadily increasing since the 1980s and is mediated by β-lactamase genes, with the extended-spectrum β-lactamase (ESBL) *bla*_CTX−M_ genes being the most frequent type worldwide [4, 5]. β -Lactamase genes can be grouped into 3 groups (1, 2, and 3) according to their functional classification and clinical substrate and inhibitor profiles as outlined by Bush and Jacoby, 2009 [6]. Key differences between the functional groups can help direct clinical intervention. For example, AmpC β-lactamases in group 1 use 3rd generation cephalosporins as their substrate and are not inhibited by clavulanic acid. ESBLs in group 2 are able to hydrolyze penicillin, narrow-spectrum and 3rd generation cephalosporins and monobactams but remain susceptible to clavulanic acid [4]. Due to the proven efficacy in killing a wide range of ESBL producing bacteria, medical professionals have increasingly relied on carbapenem antibiotics to treat extended-spectrum β-lactam resistant infections since their introduction to the market in 1985 [7]. Unfortunately, resistance to carbapenems mediated by carbapenemase genes has also developed with an increase in prevalence from 1.2 to 4.2% between 2001 and 2011 in the US [8]. This increase comes at a great cost in both clinical and financial outcomes. Carbapenem-resistant *Enterobacteriaceae* among hospitalized patients is associated with a significantly higher risk of overall mortality compared to those with carbapenem-susceptible infections [9, 10]. The economic burden of carbapenem resistant *Enterobacterales* (CRE) infections is estimated to be $66,031 for hospitals for a single infection and up to $275 million with an incidence rate of 2.93 infected people per 100,000 in the USA [11].

Carbapenemase and ESBL genes occur in a variety of species but are most prominent in *Enterobacterales* such as *Klebsiella pneumoniae*, *E. coli* and *Enterobacter* spp. [12]. Many AMR genes are commonly found on mobile genetic elements such as extrachromosomal, self-replicating plasmids which are widespread drivers of acquired resistance through mechanisms such as horizontal gene transfer. Under antimicrobial pressure, these plasmid-borne AMR genes are quickly spread between microbiota due to the immediate advantage to their bacterial host [13]. Specific plasmid replicon or incompatibility types, such as the IncF family, have been associated with widespread dissemination of AMR genes due to the conferred selective phenotype [14]. Horizontal transfer of AMR genes on these mobile genetic elements to chromosomes can result in clonal expansion and rapid dissemination [15]. Due to the inability of unique plasmid replicon types to coexist stably in a bacterial line, also known as plasmid exclusion, understanding which plasmid types are contributing to AMR circulation informs the epidemiology and risk of transmission of plasmid-mediated AMR spread in an area.

Certain clonal groups within these species, identified by their multi locus sequence type (ST), have become known as “pandemic strains”, contributing to worldwide dissemination of resistance through sustained clonal expansion. For example, the *E. coli* ST131 is associated with extraintestinal infections and is most well-known for community onset of antimicrobial resistant infections across the globe [16]. Despite its prominence as a clonal group, in depth studies have shown that the success of AMR diversity in ST131 originated from chromosomal acquisition and integration of resistance genes from mobile genetic elements [17]. Other emerging pandemic sequence types have also been identified such as ST69, ST73 and ST95 defined by their ability to cause human extraintestinal infections [18]. Gaining a better understanding of plasmid mediated resistance is imperative to better understand the risk of dissemination to new clonal sequence types and mitigate spread in an outbreak setting.

Although national support for surveillance of multidrug resistant infections exists in the United States, such as the CDC Antibiotic Resistance Laboratory Network (ARLN), individual states determine which diseases and conditions are legally reportable. In 2015, only eight states required that state providers report cephalosporin and carbapenem resistant infections [19]. In 2017, the Alameda County Public Health Department issued a mandate requiring submission of all CRE isolates detected in the county. In this study, we analyzed unidirectional short-read data of 82 *E. coli* isolates collected through the Alameda County surveillance system that identified carbapenem resistant *E. coli* (CR-Ec) in medical centers over 2017 to 2019. The analysis provides insight on the dominant *E. coli* clonal groups responsible for carbapenem resistant infections in the healthcare community. The potential role of plasmids in the dissemination of high risk ARGs is discussed as well as the benefits of population-level data in regional surveillance.

## Methods

### Sample isolation

The Alameda County Public Health Laboratory (ACPHL) works with hospitals, long-term care facilities and clinics to screen isolates for known and emerging threats and prevent outbreaks of multidrug resistant organisms. As mandated by a 2017 Health Officer Order issued by the Alameda County Public Health Department, any carbapenem resistant *Enterobacterales* (CRE) is required to be sent to ACPHL for further analysis. The team received a total of 82 carbapenem resistant *E. coli* isolates between June 2017 and July 2019 from a variety of human clinical infections occurring in the Alameda healthcare system. Samples originated from new patients as well as recurring or persistent infections in individual patients. The majority of isolates originated from urine (57.3%), rectal swab (8.5%) and blood (7.3%) specimens. Seventy-five (91%) of the isolates were isolated from hospital settings with the remaining 8% originating in long-term acute care facilities (supplemental Table 2).

Prior to submission to ACPHL, the respective Clinical Laboratory Improvement Amendments (CLIA) certified clinical laboratories within the county of Alameda examined the bacterial isolates phenotypically for resistance using either the minimum inhibitory concentration (MIC) method or Kirby-Bauer disk diffusion method. Regardless of the isolation method, all *E. coli* isolates were defined as carbapenem resistant according to CLSI M-100 culture guidelines with CLSI breakpoints of ≥ 2 µg/mL for ertapenem and ≥ 4 µg/mL for imipenem, doripenem, and meropenem [20]. Strain ATCC25922 of *E. coli* was used as a control.

### Whole genome sequencing and assembly

Purified DNA of the bacterial isolates was individually extracted using Roche MagNA Compat Instrument (Roche, Basel, Switzerland) and was performed by ACPHL. The purified DNA was quantified by fluorimetry using Qubit 3.0 (Invitrogen, Carlsbad, CA, USA).

Whole genome sequencing library preparation was completed by ACPHL. Quantified DNA was diluted to 0.2 ng/ul, fragmented, and tagged using Nextera XT Library Preparation Kit (Illumina, San Diego, CA, USA). The Qubit 3.0 fluorometer in combination with Qubit negative and Qubit positive broad range standards were used to quantify the quality of purified Indexed Libraries. Pooled equimolar quantities of the samples were sequenced using single-end, 150 cycle reactions with a depth of 40X in a MiSeq (Illumina, San Diego, CA, USA) at ACPHL. Unicycler’s SPAdes based pipeline [21] was used to assemble the Illumina unpaired unidirectional short reads using de-novo assembly without a reference genome.

The Klebsiella pneumoniae isolate BAA-2146 (ATCC, Manassas, VA, USA) was used as a control for each sequencing run procedure, from DNA extraction though sequencing analysis.

### Genomic analysis

ACPHL identified and assigned the isolate’s species using Genomic Approximation Method for Bacterial Identification and Tracking (GAMBIT) software with a reference database of over 50,000 genome sequences [22]. The Klebsiella pneumoniae isolate BAA-2146 was used as the control organism. *E. coli* isolates underwent multi-locus sequence typing using seven housekeeping genes: *adk, fumC, gyrB, icd, mdh, purA*, and *recA*. The MLST software [23] in tandem with the PubMLST database [24] was used for this analysis. Higher resolution of genetic relatedness between CTX-producing isolates was achieved using core genome multi locus sequence typing. The Center for Genomic Epidemiology’s cgMLSTFinder 1.1 was used to run KMA [25] against an Enterobase *E. coli* cgMLST scheme containing 2513 genes [26]. A dendrogram of the resulting allele matrix was created through the T rex webserver [27] using the Neighbor Joining method [28] with a Kimura 2-Parameters substitution method and ignoring missing bases.

Contigs of assembled sequences were analyzed using the ResFinder 4.0 database [29] from the ABRicate package [30] to detect antimicrobial resistance genes. The default search parameters of 90% sequence identity with a minimum sequence overlap of 60% were used. Resistance genes were grouped according to their functional profiles as per pre-defined methods in addition to the NIH reference gene catalog [6, 27]. Mlplasmids [32] and MOB-suite [33] were used to classify contigs as either plasmid or chromosome borne. MOB-suite uses an ensemble-based approach referencing Blast and Mash Databases to identify and assemble plasmid contigs. The default parameter for *E. coli* plasmids was used. Mlplasmids uses a support-vector machine learning model that has been trained using complete *E. coli* genomes to accurately predict short-read location. The default value of 0.5 for the minimum posterior probability belonging to the plasmid class versus chromosome class was used and contigs smaller than 1000 base pairs were filtered out after analysis to ensure all contigs were annotated.

### Data analysis and visualizations

Relative abundance of resistance genes was calculated by dividing the count of one resistance gene within the ST by the total number of resistance genes present in the ST. Contigs with discordant classification between the mlplasmids and MOB-suite programs were labelled as ambiguous and excluded from the final analysis, in accordance with previous studies [34]. The cgMLST dendrogram based on the allele matrix of CTX producing isolates was visualized using iTOL version 6.4.3. Genetic distance was calculated according to previous work by dividing the number of allele differences between two isolates by the total number of shared alleles [35]. Clonal groups were categorized due to clinical relevancy as documented in previous literature [31–37]. All data manipulation and analyses were done using R Studio Software version 1.4.1073 in tandem with the following R packages: ggplot [43], dplyr [44], stringr [45], and tidyr [46].

## Results

### Sequence types and antimicrobial resistance genes

**Fig. 1:**
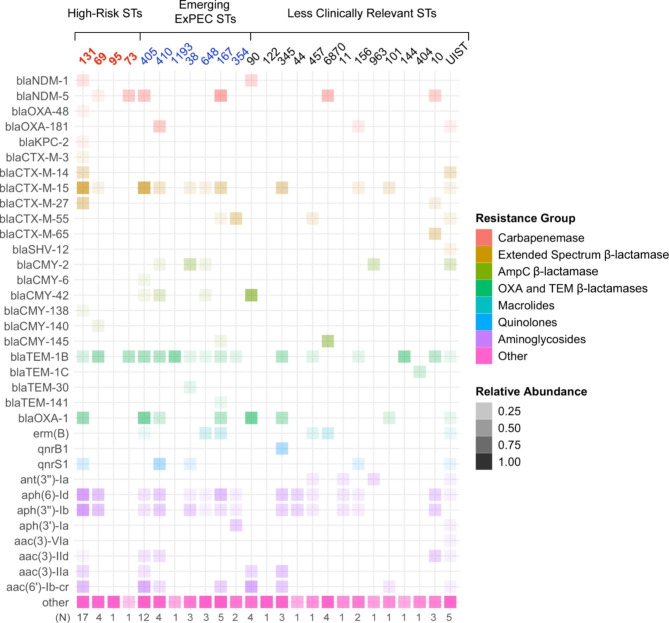
Relative abundance of antimicrobial resistance genes across *E. coli* sequence types Legend: Relative abundance is the total count of a resistance gene in the ST divided by the total number of resistance genes in the ST. The mean value of relative abundance was calculated for isolates within the same ST and resistance group. Clonal groups were categorized due to clinical relevancy as documented in previous literature [31–37]

The collection of carbapenem resistant *E. coli* presented a wide diversity of ST’s based on MLST analysis. Of the 82 isolates sequenced, 25 different ST’s were identified, four of which are known to be high risk (Fig. 1). About 64% (53/82) of the isolates belonged to either a high-risk sequence type or an emerging high risk sequence type. The most common of the sequence types was ST131, representing 20% (17/82) of the total isolates. The emerging ST405 was the second most common with twelve isolates. Five isolates did not have a MLST sequence type assigned, but did have a cgMLST, suggesting they may belong to a previously unidentified ST.

Carbapenemase genes were not detected in 55 of the 82 isolates, including the emerging sequence types such as ST648, ST1193 and ST38. The *bla*_NDM−5_ carbapenemase gene was highly prevalent in ST73, ST405, ST167 and ST6870. ST131 encompassed a large share of *bla*_OXA−1_ genes in addition to ST405, ST410 and ST167. In contrast, most isolates (72/82) contained at least one β-lactamase gene. The most widespread β-lactamase gene was *bla*_TEM−1B_, which was detected across 16 sequence types. ST131 contained the largest diversity of resistance genes and included both broad and extended spectrum β-lactamase TEM, OXA, NDM, CTX and CMY genes in addition to various aminoglycoside and quinolone resistance genes. Many isolates contained some combination of β-lactamase genes (Table 1). More than one fifth (18, 22%) of isolates carried an ESBL gene in addition to a OXA or TEM β-lactamase while about three of the isolates (4%) carried β-lactamase genes from all four categories.

**Table 1:** Frequency of β-lactamase Resistance Gene Co-Carriage Across Isolates

β-lactamase Gene Repertoire	Number of Isolates	Percent of total Isolates
Extended Spectrum β-lactamase, OXA and TEM β-lactamases	18	21.95
Extended Spectrum β-lactamase	13	15.85
Extended Spectrum β-lactamase, Carbapenemase, OXA and TEM β-lactamases	8	9.76
AmpC β-lactamase	5	6.10
OXA and TEM β-lactamases	5	6.10
AmpC β-lactamase, Carbapenemase	4	4.88
AmpC β-lactamase, OXA and TEM β-lactamases	4	4.88
AmpC β-lactamase, Carbapenemase, OXA and TEM β-lactamases	3	3.66
AmpC β-lactamase, Extended Spectrum β-lactamase, Carbapenemase, OXA and TEM β-lactamases	3	3.66
Carbapenemase, OXA and TEM β-lactamases	3	3.66
Carbapenemase	2	2.44
Extended Spectrum β-lactamase, Carbapenemase	2	2.44
AmpC β-lactamase, Extended Spectrum β-lactamase	1	1.22
AmpC β-lactamase, Extended Spectrum β-lactamase, OXA and TEM β-lactamases	1	1.22
AmpC β-lactamase, OXA and TEM β-lactamases, Carbapenemase	1	1.22

### Plasmid bound resistance genes

**Fig. 2:**
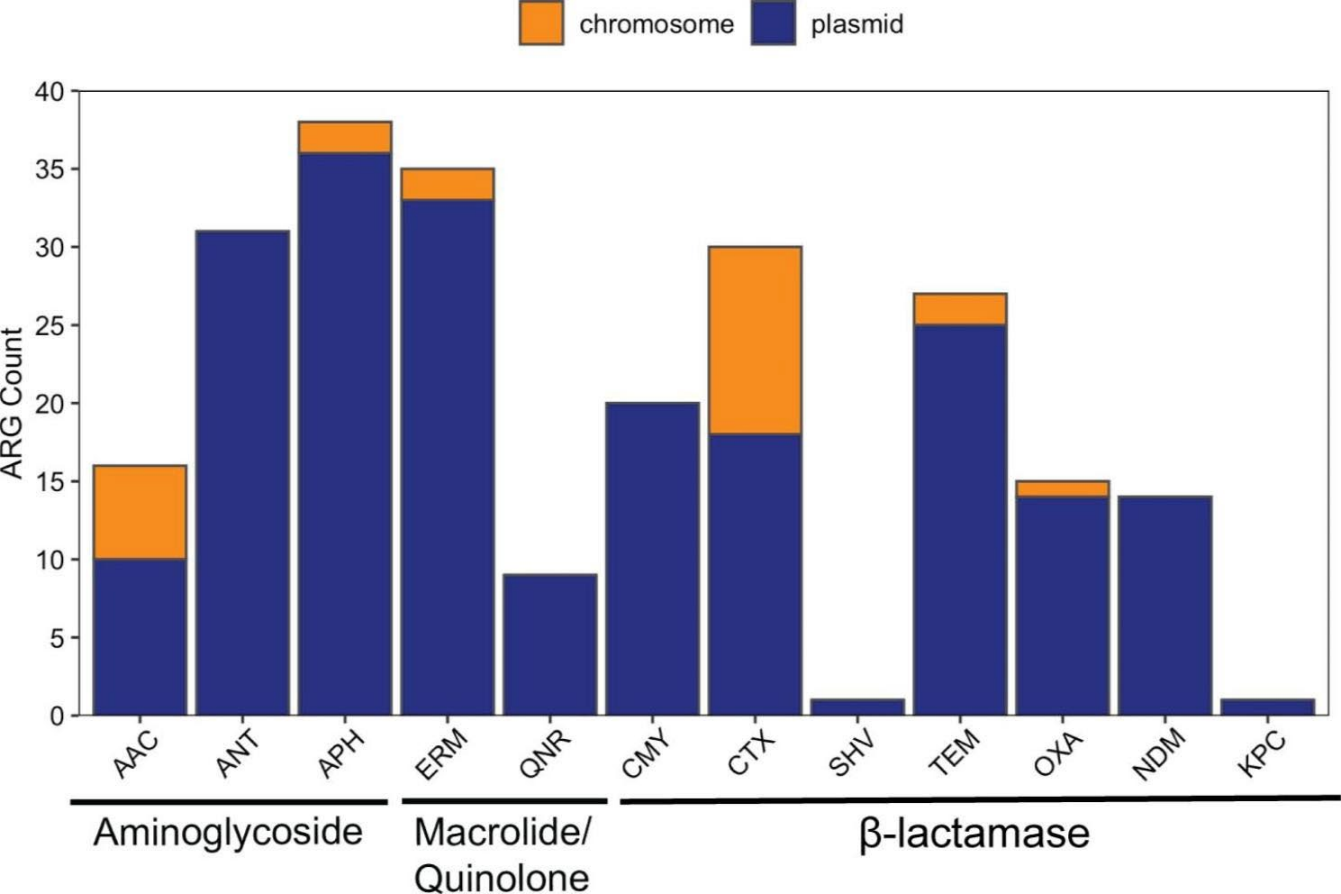
Proportion of plasmid bound antimicrobial resistance genes (ARGs). Legend: Antimicrobial resistance genes were detected using the ResFinder database in ABRicate and contigs were determined to be plasmid or chromosomal using mlplasmids v1.0 and MOB-suite v3.0.3

The majority of β-lactamase genes were located on plasmid contigs, as defined previously [16, 17]. Over half of CMY, NDM, OXA, and TEM genes were definitively plasmid-borne (Fig. 2). The one KPC gene and the one SHV gene that were detected existed only on plasmid contigs. The *bla*_CTX−M_ genes were the most common ESBL genes and a little over half (18/30) of the *bla*_CTX−M_ genes were identified as plasmid-borne. Eleven antibiotic resistance genes defined as current threats among pathogens were detected and ranged across the aminoglycoside, β-lactam, macrolide-lincosamide-streptogramin (MLS), and trimethoprim families (supplemental Table 1). All of trimethoprim genes were plasmid bound, while the majority of β-lactam (62-100%), MLS (67%), and aminoglycoside (88%) genes were also plasmid bound.

Although the present methods were not able to indicate whether multiple contigs within an isolate mapped to the same plasmid, grouping of multiple resistance genes on the same plasmid contig was observed. As shown in Tables 2, the majority (76%) of β-lactamase genes presented individually on plasmid contigs. Twenty-one (23%) of the contigs containing a carbapenemase or a β-lactamase gene were colocalized with an aminoglycoside resistance gene. The most common gene cluster contained the OXA β-lactamase gene, *bla*_OXA−1_, together with the aminoglycoside aac(6’)-Ib-cr gene, which was present in six isolates. One isolate from ST345 had a plasmid contig containing two β-lactamase genes: ESBL gene, *bla*_CTX−M−15_, and TEM β-lactamase gene, *bla*_TEM−1B_. No other contigs containing multiple carbapenemase genes or a combination of β-lactamase and carbapenemase genes were detected despite co-carriage of β-lactamase genes in most isolates.

**Table 2:** Distribution Antimicrobial Gene Clusters Identified on Plasmid Contigs

Resistance Group	Resistance Type	AMR Gene Cluster	Contig Total	Sequence Types (n)
β-lactamase	β-lactamase	*bla*_CTX−M−15_	11	131 (3), 167 (2), 405 (2), 410 (2), 69, 345
		*bla*_CTX−M−15,_ *bla*_TEM−1B_	1	345
		*bla*_CTX−M−27_	1	10
		*bla*_CTX−M−55_	3	354 (2), UIST*
		*bla*_CTX−M−65_	1	10
		*bla*_CMY−138_	1	131
		*bla*_CMY−140_	1	69
		*bla*_CMY−145_	4	6870 (3), 167
		*bla*_CMY−2_	4	648, 963, 38, UIST*
		*bla*_CMY−42_	8	90 (4), 410 (2), 648, 405
		*bla*_CMY−6_	1	405
		*bla*_OXA−1_	1	405
		*bla*_SHV−12_	1	UIST*
		*bla*_TEM−1B_	14	410 (2), 131 (2), 10 (2), 648, 69, 345, 156, 1193, 38, 144, 73, 405
		*bla*_TEM−1 C_	1	404
		*bla*_TEM−30_	1	38
	β-lactamase, Quinolone	*bla*_CTX−M−15,_ qnrS1	1	38
	β-lactamase, Aminoglycoside	*bla*_TEM−1B,_ rmtB	3	167 (2), 405
		*bla*_OXA−1_, aac’(6’)-Ib-cr	6	345 (3), 90 (2), 167 (2)
	β-lactamase, Aminoglycoside, Sulfonamide	*bla*_TEM−1B,_ aph(3’’)-Ib, aph(6)-Id, sul2	5	69 (2), 345, 354, 457
	β-lactamase, Aminoglycoside, Macrolide, Sulfonamide	*bla*_CMY−2_, aac(3)-VIa, ant(3’’)-Ia, mef(C), mph(G), sul1, sul2	1	UIST*
Carbapenemase	Carbapenemase	*bla*_KPC−2_	1	131
		*bla*_NDM−1_	2	131
		*bla*_NDM−5_	6	6870 (3), 167, 405, 10
		*bla*_OXA−48_	1	131
	Carbapenemase, Quinolone	*bla*_OXA−181_, qnrS1	5	410 (3), 156, UIST*
	Carbapenemase, Aminoglycoside, Sulfonamide, Trimethoprim	*bla*_NDM−1_, aadA2, sul1, dfrA12	2	90
		*bla*_NDM−5_, aadA2, sul1, dfrA12	3	167 (2), 405
		*bla*_NDM−5_, aadA5, sul1, dfrA17	1	405

*Unidentified Sequence Typ**e**

### Clonal relationship of CTX-producing isolates

**Fig. 3:**
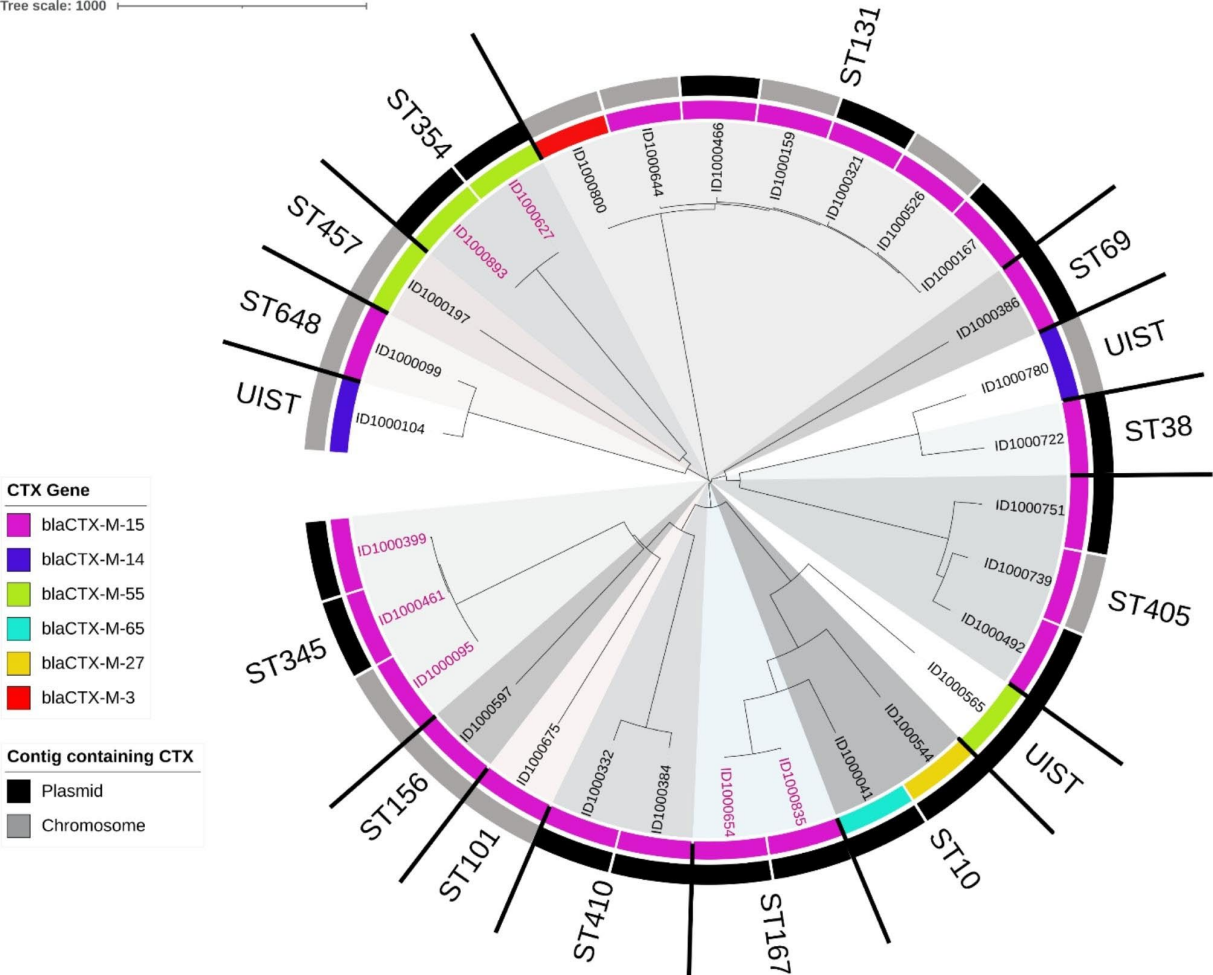
Neighbor-joining tree of 30 CTX-producing *E. coli* isolates based on a cgMLST EnteroBase scheme including 2513 genes Legend: The tree was built using cgMLSTFinder v.1.1 and T-Rex webserver and visualized with iTOL v.6.4.3. Different MLST clusters are highlighted in various shades of grey. Isolate pairs with the same cgMLST sequence type are highlighted in pink. The color-coded rings represent the contig type containing the CTX gene as called by mlplasmids and MOB-suite (outer ring) and the CTX gene (inner ring)

As shown in Table 3, ST’s determined by MLST continued to group together after cgMLST analysis. The widest diversity was observed in ST10 with allele differences ranging from 0 to 682. The most related isolates belonged to ST131 with allele differences ranging from 4 to 61. Nine of the twenty-one ESBL *bla*_CTX−M−15_ genes belonged to ST131 and ST405 and were evenly distributed between being plasmid-borne and chromosome-borne (Fig. 3). The *bla*_CTX−M−55_ genes were similarly found on both plasmid and chromosomal contigs. The *bla*_CTX−M−65_ and *bla*_CTX−M−27_ genes were exclusively plasmid-borne while *bla*_CTX−M−14_ and *bla*_CTX−M−3_ genes were exclusively chromosomal.

**Table 3:** Genetic Distance of Clonally Related CTX-producing *E. coli* Isolates

Isolate	ST	cgST	Allele Differences	Genetic Distance*	Sample Source	Localization of CTX gene
ID1000095 ID1000399 ID1000461	345	26,123	2	0.0008	Same	Chromosome Plasmid Plasmid
ID1000627 ID1000893	354	33,210	1	0.0004	Same	Plasmid Plasmid
ID1000654 ID1000835	167	141,806	7	0.0029	Different	Plasmid Plasmid

*Calculated by dividing allele differences by number of shared alleles

Clonally related isolate pairs belonging to the same core genome ST were identified, including: ST354, ST345, and ST167 (highlighted in Table 3). Genetic distance calculations suggested the pairs were epidemiologically related within a threshold of 0.0105 (Table 3). The clonal pair in ST354, contained CTX-M-55 genes while the clonal pairs in ST167 and ST345 contained CTX-M-15 genes. Clonally related pairs in ST345 contained both a plasmid and chromosomal localization of the *bla*_CTX−M−15_ gene and were isolated from the same individual.

## Discussion

Antibiotic resistance is a critical threat to patients in various healthcare facilities across the country with regard to treatment options, outcomes, and costs. Despite its increasing complexity, the majority of local surveillance focuses on phenotypic resistance rather than the wide variety of genetic elements influencing resistance transmission. Incidence reporting of resistant infections provides an incomplete picture of the threat of resistance as mobile genetic elements, such as plasmids, can transmit resistance to previously susceptible strains [47]. Despite its relevance, routine genomic surveillance remains challenging due to a lack of funding for local laboratories and organized higher-level guidance. For this reason, short-read sequencing is becoming an increasingly popular method for genomic surveillance due to its speed and affordability.

In the present study, we used short-read whole genome sequences of 82 carbapenem resistant *E. coli* to investigate the biological mechanisms driving clinically important AMR in Alameda County, California. Our analysis offers insight into the genomic characteristics of dominant clonal groups, the potential role of plasmids in the regional dissemination of ARGs and provides support for the importance of population-level reporting to local health departments.

### Antimicrobial resistance across sequence types

The isolates presented a diverse profile of high risk and emerging STs, illustrating the growing expanse of potential pandemic lineages in Alameda County. The prevalence of ST131 (20%) in Alameda County is in alignment with country-level findings. A recent report found ST131 made up about 30% of carbapenem resistant *E. coli* infections in the US from 2011 to 2015 [48]. In contrast, a large share of isolates in Alameda County belonged to the emerging ST405 also known to cause extraintestinal infections, but not detected in the nationwide survey. Few studies have detailed the worldwide prevalence and evolution of ST405 but a recent increase in prominence has been detected in certain countries outside of the US [32, 43]. Its high prevalence in Alameda County suggests it may develop into a pandemic strain and highlights the importance of regional surveillance.

Despite being carbapenem resistant phenotypically, only 30% of the isolates contained carbapenemase genes, which suggests these organisms are mediating resistance through alternative mechanisms. Overexpression of ESBL and AmpC β-lactamases, such as the *bla*_CTX_ and *bla*_CMY_ genes, coupled with a loss of porin function have been shown to confer resistance to carbapenems [22, 23]. Although this study’s focus is on resistance genes, the diverse repertoire of various β-lactamase genes across isolates suggests this may be a possible mechanism mediating phenotypic carbapenem resistance. Although CTX-M enzymes are the most globally dominant ESBL, the widespread prevalence of *bla*_TEM−1B_ across sequence types suggests this gene may be regionally important and an artifact of the use of extended spectrum penicillin’s and first generation cephalosporins in the healthcare community. Although high risk ST’s contained the largest share of carbapenemase and ESBL genes, many of the non-pandemic clonal groups such as ST90, ST6870, and ST354 also demonstrated a relatively high prevalence. Notably, the high-risk clonal group ST95, did not contain any carbapenemase or β-lactamase genes. This is in alignment with recent research suggesting that plasmid exclusion due to incompatibility results in lower resistance accumulation [52].

### Plasmid localization of resistance genes

The presence of repetitive DNA elements near carbapenemase genes most likely explains the high frequency of resistance genes located on individual contigs. Short-read only assembly graphs are broken by repetitive DNA elements, such as transposon-related sequences, resulting in an incomplete assembly of plasmid elements [53]. Despite this multiple gene clusters were observed including one containing the β-lactamase genes, *bla*_TEM−1B_ and *bla*_CTX−M−15_ in an isolate belonging to ST345. Co-carriage of these genes is often associated with the highly diverse IncF plasmids [54]. The CTX genes are also often colocalized with OXA genes on IncF plasmids and multiple isolates showed co-carriage of the two genes, but they were not mapped to the same contig. IncF plasmids are naturally found in the human fecal flora but have potential to act as epidemic plasmids due to their ability to acquire resistance genes and quickly disseminate [13]. Additional spatial analysis supplemented by long read sequencing, would help determine whether various β-lactamase genes are colocalized to the same plasmid within individual isolates and establish the resulting potential of replicon dissemination.

Our isolates also showed a high prevalence of eleven high risk antimicrobial resistance genes from four ARG families (aminoglycoside, β-lactam, MLS and trimethoprim) known to cause problems in hospitals and often found on mobile genetic elements [55]. In alignment with previous literature, the majority of these high-risk resistance genes were plasmid-bound, including all trimethoprim resistance genes. Six of the plasmid-bound trimethoprim resistance genes were detected on contigs that also contained carbapenemase, aminoglycoside and sulfonamide genes. Older generation antibiotics such as trimethoprim have been used as an alternative treatment for carbapenem resistant infections [56]. The plasmid co-localization of trimethoprim resistance genes with carbapenemase genes is of concern and suggests circulation of high-risk multi-drug resistant plasmids in Alameda County.

The *bla*_CTX−M_ genes are known to exist more frequently on plasmids but have increasingly been observed on chromosomes [57]. The isolates in this dataset showed an equal distribution of the two sites. Epidemiologically related isolate pairs demonstrated both a plasmid and chromosome version of the *bla*_CTX−M−15_ gene, suggesting recent chromosomal integration of plasmid bound *bla*_CTX−M−15_ genes or vice versa. This finding highlights the rapid evolution of resistance gene localization, even within a single patient. The movement of the *bla*_CTX−M−15_ gene is of additional interest as a recent study demonstrated differential resistance rates when located on a plasmid compared to the chromosome and plasmid mediated spread may influence the rate of dissemination in an outbreak setting [58].

Dissemination of *bla*_CTX−M−15_ is traditionally attributed to the success of clonal species containing AMR plasmids, especially ST131 clones and IncF plasmids [57]. Our results support this theory, as the *bla*_CTX−M−15_ gene was prominent in select clonal groups with varying plasmid associations. However, without long read sequencing to conduct comparative analysis, the full extent of the plasmids role in this dissemination cannot be established.

### Limitations and applications

Whole genome sequencing has transformed resistome surveillance. Short-read sequencing is currently the most commonly used form of next generation sequencing due to its low cost and high accuracy. Despite its widespread use, the repetitive DNA elements of plasmids continue to prevent full spatial reconstruction from short-read sequences, limiting insight into plasmid dynamics and structure. We were not able to fully establish the diversity and frequency of antimicrobial resistance genes that are colocalized to the same plasmid, limiting our insight to the full risk of clinically relevant resistance plasmids circulating in the county. We were not able to infer genetic characteristics that may be responsible for plasmid propagation and gene transfer. For example, transposon sequences that aid in lateral resistance gene movement from chromosome to plasmid or vice versa could not be structurally reconstructed. In alignment with previous analyses, our supplemental analyses also indicated that our current methods, although accurate, provided limited insight into plasmid contigs replicon type (data not shown) [59]. Without broad replicon typing, it is impossible to assess the risk of possible epidemic plasmids such as the IncF plasmids [60]. A hybrid assembly using short-read and supplemental long-read sequences has been shown to be the most comprehensive method to assess these risks [61].

This study was limited to clinical isolates that were screened for carbapenem resistance due to a lack of response to carbapenem antibiotics in patients. Inference on the risks of resistance genes and circulating sequence types in the medical system may be biased as it does not account for other potential sources of carriage in the healthcare community such as asymptomatically colonized staff and patients. It also does not consider environmental reservoirs such as the healthcare environment and wastewater reservoirs which are known drivers in AMR transmission [34, 35]. A more robust screening program for asymptomatic colonization would need to be implemented in order to investigate the prevalent clonal groups and resistance genes circulating in the larger community, outside of an infection setting.

There is a growing movement for national laboratory capacity for WGS in AMR monitoring through federally coordinated networks with state agencies and universities. Although phenotypic methods are the main screening tool in healthcare site, information on microbial factors that can influence the outcome of an infection can be overlooked. Depending on the antibiotic panel used in the phenotypic screening, insight on the frequency of co-circulation of resistance and feasible treatment options may be limited [64, 65]. For example, the co-occurrence of carbapenemase genes with trimethoprim and aminoglycoside genes suggests these two antibiotics are not suitable alternatives in this population. Further, the presence of multidrug resistant plasmid fragments present in this study supports the need for immediate infection control protocols in hospital sites across Alameda County, in order to prevent rapid spread of carbapenem resistance to susceptible species. This study demonstrates that short-read screening can be incorporated in tandem with phenotypic analyses into routine local surveillance to evaluate the risk of CRE in healthcare settings. Findings from this study can be applied to environmental and animal genomic surveillance in the area to expand on the microbial risk analysis in the community at large.

## Conclusion

Alameda County, California is one of the few counties in the United States that mandates carbapenem resistant *Enterobacterales* isolates be submitted to the local public health department for further analyses. Our genomic analyses were able to identify a range of AMR genes circulating in carbapenem resistant *E. coli*, the majority of which were detected on plasmids. Our results highlight several clonal groups unique to Alameda County, CA, in addition to the traditional sequence types such as ST131, ST69, ST95 and ST73. Co-localization of high-risk trimethoprim and carbapenemase and ESBL resistance genes on plasmid contigs indicate circulation of high-risk multidrug resistant plasmids in the population with the potential to propagate to these novel clonal groups and expand rapidly. Movement of a *bla*_*CTX*_ gene between plasmid and chromosome localizations highlights the rapidly changing landscape of AMR in hospitalized patients and the need for comprehensive surveillance. Together, our results illustrate the benefits of, and provide support for, routine local genomic surveillance in order to fully understand population-level dynamics of mobile genetic elements facilitating the dissemination of antimicrobial resistance in the healthcare community.

## Electronic supplementary material

Below is the link to the electronic supplementary material.

Supplementary Material 1

Supplementary Material 2

## Data Availability

The datasets and code supporting the conclusions of this article are available in the GitHub repository: https://github.com/niwalas/Alameda_CRE_2022. Assembled genome sequences are publicly available on GenBank. The bio project can be accessed at the following link: https://www.ncbi.nlm.nih.gov/bioproject?LinkName=nuccore_bioproject&from_uid=2355685487.
