# Supplementary material for: The role of plasmids in carbapenem resistant E. coli in Alameda County, California
Source: BMC Microbiol. 2023 May 22;23:147. doi: 10.1186/s12866-023-02900-2 (PMC10201492; doi:10.1186/s12866-023-02900-2)
Supplement: Supplementary file 1 — Supplementary Material 1 [file 12866_2023_2900_MOESM1_ESM.docx]

**Supplemental Table 1:**

| Antibiotic Class/High risk antibiotic resistance gene | Total count of ARG (n) | Percent identified on Plasmid* |
| --- | --- | --- |
| Aminoglycoside |  |  |
| *aac(6’)-Ib-cr* | 23 | 87.5 |
| Beta-Lactam |  |  |
| *blaCMY-6* | 1 | 100 |
| *blaCTX-M-15* | 29 | 61.9 |
| *blaCTX-M-55* | 5 | 75 |
| *blaKPC-2* | 1 | 100 |
| *blaNDM-5* | 16 | 100 |
| Trimethoprim |  |  |
| *dfrA5* | 1 | 100 |
| *dfrA12* | 19 | 100 |
| *dfrA14* | 9 | 100 |
| *dfrA17* | 26 | 100 |
| Macrolide-lincosamide-streptogramin (MLS) |  |  |
| *erm(B)* | 9 | 66.7 |

*Proportion among genes with non-ambiguous contig calls.
