# Supplementary material for: The role of plasmids in carbapenem resistant E. coli in Alameda County, California
Source: BMC Microbiol. 2023 May 22;23:147. doi: 10.1186/s12866-023-02900-2 (PMC10201492; doi:10.1186/s12866-023-02900-2)
Supplement: Supplementary file 2 — Supplementary Material 2 [file 12866_2023_2900_MOESM2_ESM.docx]

Supplemental Table 2:

| Collection Category | Specimen Source | Healthcare setting | Count (n) | Proportion of total isolates (%) |
| --- | --- | --- | --- | --- |
| Blood | Blood | Hospital | 6 | 7.3% |
| Enteric | Stool | Hospital | 2 | 2.4% |
|  | Rectal Swab | LTAC* | 7 | 8.5% |
|  | Rectal Swab | Hospital | 4 | 4.9% |
| Tissue | Abscess | Hospital | 3 | 3.7% |
|  | Jackson-Pratt drainage | Hospital | 1 | 1.2% |
|  | Sacral tissue | Hospital | 1 | 1.2% |
| Urine | Urine | Hospital | 47 | 57.3% |
| Body fluids | Sputum | Hospital | 3 | 3.7% |
|  | Peritoneal Fluid | Hospital | 1 | 1.2% |
|  | Dialyste Fluid | Hospital | 1 | 1.2% |
| Unknown | Unknown | Hospital | 4 | 4.9% |
| Other | Bile | Hospital | 1 | 1.2% |
|  | Right Kidney | Hospital | 1 | 1.2% |

*Long term acute care facility
